# Supplementary material for: Third-party punishment by preverbal infants
Source: Nat Hum Behav. 2022 Jun 9;6(9):1234–42. doi: 10.1038/s41562-022-01354-2 (PMC9489529; doi:10.1038/s41562-022-01354-2)
Supplement: Supplementary file 1 — Supplementary Figs. 1–3 and Tables 1–13. [file 41562_2022_1354_MOESM1_ESM.pdf]

nature portfolio

https://doi.org/10.1038/s41562-022-01354-2

Supplementary information

Third-party punishment by preverbal infants

In the format provided by the authors and unedited

Third-party punishment by preverbal infants

Yasuhiro Kanakogi[1*†], Michiko Miyazaki[2†], Hideyuki Takahashi[3†], Hiroki Yamamoto[1, 4], Tessei Kobayashi[5], and Kazuo Hiraki[6]

[1]Graduate School of Human Sciences, Osaka University

[2]Faculty of Social Information Studies, Otsuma Women's University

[3]Graduate School of Engineering Science, Osaka University

[4]Graduate School of Letters, Kyoto University

[5]NTT Communication Science Laboratories

[6]Graduate School of Arts and Sciences, The University of Tokyo

*Correspondence to: Yasuhiro Kanakogi, Graduate School of Human Sciences, Osaka University, 1-2, Yamadaoka, Suita, Osaka 565-0871, Japan. Tel: +81-6-6879-8043. E-mail: y-kanakogi@hus.osaka-u.ac.jp.

[†]Equally contributed to this work.

Supplementary Information

To check whether our sampling design had sufficient power to detect the effect of test type, we computed simulation-based power, given the actual sample size, and the theoretically expected effect size. We randomly generated 100 samples based on a theoretically expected effect size while setting various values for the magnitude of individual difference. Subsequently, we fitted a statistical model to the 100 samples to evaluate the extent to which the 95% credible interval (CI) of the test type effect excluded zero. We generated a sample based on the following model formulas:

$$Y_{ij} \sim Bernoulli(q_{ij}) \quad (1)$$

$$logit(q_{ij}) = \beta_0 + (\beta_1 + r_{2i}) * X_1 + \beta_2 * j + r_{1i} \quad (2)$$

$$r_{1i} \sim Normal(0, \sigma_1) \quad (3)$$

$$r_{2i} \sim Normal(0, \sigma_2) \quad (4)$$

Let us assume that the infant i's selective looking at the antisocial agent at trial number j Y_{ij} follows a Bernoulli distribution of parameter q_{ij}(formula 1). Infant's selective looking at an aggressor or a causer was treated as 1, otherwise as 0. The logit link function is applied for q_{ij} and the linear predictor is defined as formula 2. The random intercept r_{1i} represents individual difference in the tendency to selectively look at antisocial agents, and we assumed that it follows normal distribution with mean 0 and standard deviation σ_1 (formula 3). The random slope r_{2i} represents the individual difference in the effect of test type, and we assumed that it follows the normal distribution with mean 0 and standard deviation σ_2 (formula 4). Sample size was set to 24 (see Methods in Main Text). The explanatory variable X_1 represents test type (pretest/posttest) and it was dummy coded with the reference category 'pretest'. β_0 is the intercept, β_1 is the coefficient parameter of test type, and β_2 is the coefficient parameter of trial number. Since we set β_2 to 0 in the simulation, population-level parameter q_j in the pretest is represented as $inv\_logit(\beta_0)$ and q_j in the posttest is represented as $inv\_logit(\beta_0 + \beta_1)$.

As a general setting for generating samples, we set population level q_j in the pretest as 0.5 (chance level) and population level q_j in the posttest as 0.68. The value of q_j in the posttest was determined based on a previous meta-analysis study[1] which estimated infants' preferences between a prosocial and an antisocial agent. Since we could not know the magnitude of individual difference (σ_1 and σ_2) *a priori*, we randomly generated 100 samples with a combination of σ_1 and σ_2, while setting each of σ_1 and σ_2 from 0.1 to 2 in increments of 0.1.

For each of the 100 samples obtained from a given pair of σ_1 and σ_2, we fitted the model with the main effects of test type, trial number, and their interaction. We set participant identity as a random intercept. We also included all possible random slopes within the participants and correlations in the model.

Model fitting was implemented using *brms* package[2, 3] in R. 4.0.3[4]. *brms* package is a high-level interface to Stan[5], which is a platform for full Bayesian inference with the Markov chain Monte Carlo (MCMC) sampling. As a general setting for MCMC sampling, iterations were set to 2000, and burn in samples were set to 1000, with the number of chains set to four. For model fitting, we set *t* distribution with degree of freedom 3 and scale 2.5 as a prior distribution of an intercept and standard deviation of random effects. After each model fitting, we checked if the model was converged based on the Rhat values of all parameters. If the model was converged, we calculated 95% CI of the test type effect β_1, and checked whether the 95% CI included zero. For each of the 100 samples obtained from a given pair of σ_1 and σ_2, we calculated the proportion of 95% CI that excluded zero in the number of times that model convergence was confirmed. Although we understand that there is a mismatch between the inference based on Bayes factor and the parameter estimation[6], for the sake of simplicity, we treated this value as a simulated power given the actual sample size and the theoretically expected effect size.

The simulated powers given the actual sample size and the theoretically expected effect size are shown in Supplementary Figure 2a. Whether the simulated power was larger than 0.8 is shown in Supplementary Figure 2b. As long as σ_2 (y-axis) was less than 0.8, simulated powers were above 0.8 in a wide range of σ_1 (x-axis). However, when σ_2 was large, simulated powers were likely to decrease.

The black dot in Supplementary Figure 2 represents a pair of estimated SD of random intercept and SD of random slope for the test type effect in each experiment (posterior median). Only in Experiment 3, the estimated pairs of SD of random intercepts and random slopes were located in the region where simulation-based power was above 0.8. This result suggests that if we suppose our sample was generated from a

theoretically expected effect size, our sampling design had sufficient power only when the individual difference was small, as reflected in Experiment 3. Our sampling design would be underpowered, especially when the individual difference of test type effect was large as reflected in Experiment 1. Although we could not know the magnitude of individual difference of test type a priori in this study, it is advisable to select a larger sample size when conducting a similar paradigm in the future.

References

1. Margoni, F. & Surian, L. Infants' evaluation of prosocial and antisocial agents: A meta-analysis. *Dev. Psychol.* **54**, 1445–1455 (2018). https://doi.org/10.1037/dev0000538
2. Bürkner, P-C. Brms: An R Package for Bayesian multilevel models using Stan. *J. Stat. Softw.* **80**, 1–28 (2017). https://doi.org/10.18637/jss.v080.i01
3. Bürkner, P-C. Advanced Bayesian multilevel modeling with the R package brms. *R J.* **10**, 395–411 (2018). https://doi.org/10.32614/RJ-2018-017
4. R Core Team. *R: A Language and Environment for Statistical Computing* (R Foundation for Statistical Computing, 2020).
5. Carpenter, B., Gelman, A., Hoffman, M. D., Lee, D., Goodrich, B., Betancourt, M., Brubaker, M., Guo, J., Li, P. & Riddell. Stan: A probabilistic programming language. *J. Stat. Softw.* **76**, 1–32 (2017). https://doi.org/10.18637/jss.v076.i01
6. Tendeiro, J. N. & Kiers, H. A. A review of issues about null hypothesis Bayesian testing. *Psychol. Methods* **24**, 774–795 (2019).

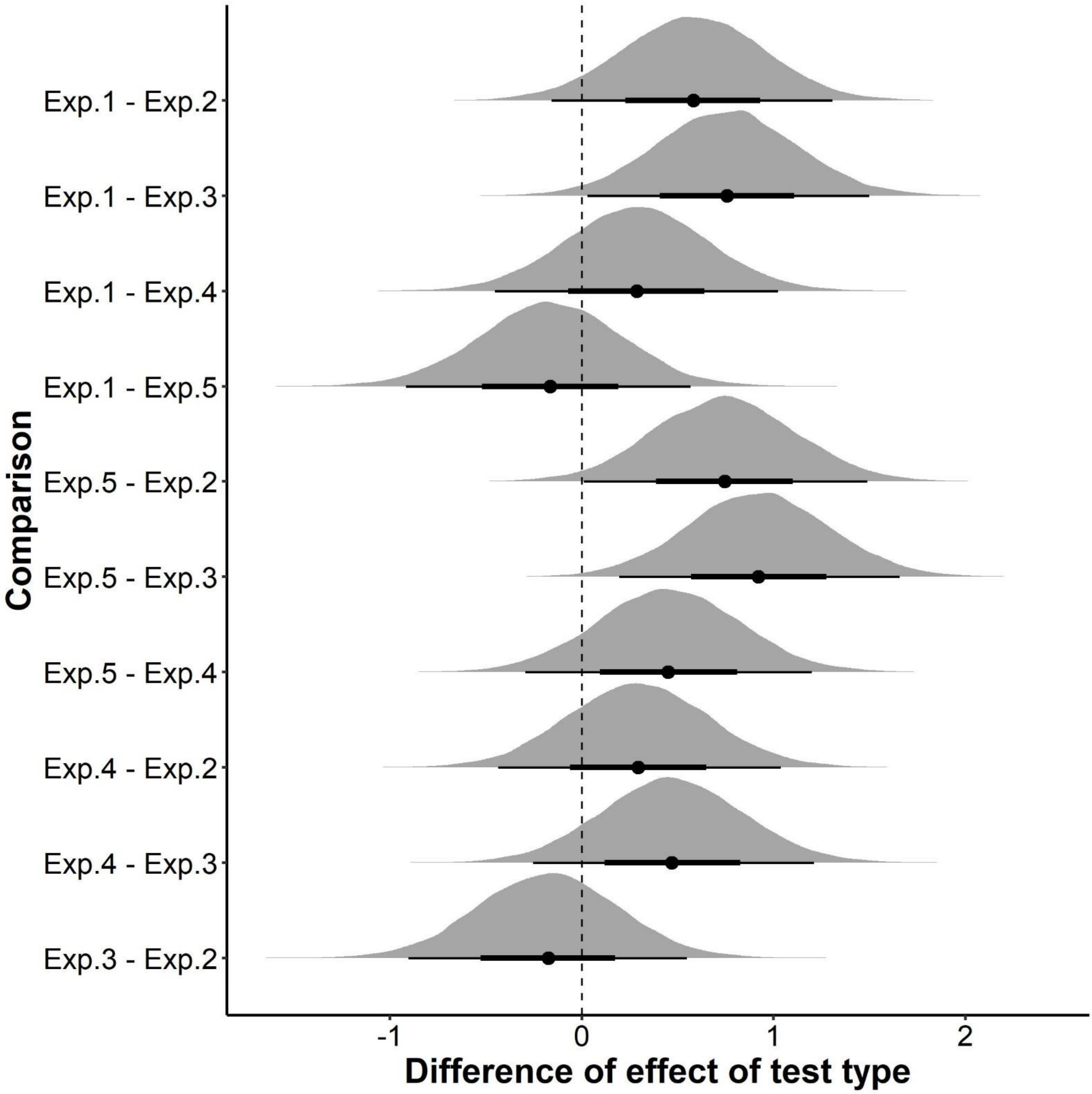

Supplementary Figure 1. Comparison of effect size of test type between experiments. The density plot shows the posterior distribution of differences in the effect of test type between each experiment. Thin error bars and thick error bars represent 95% and 66% credible intervals, respectively. The point in an error bar represents the posterior median. Twenty-four participants were allocated for each experiment, and we examined n=120 participants throughout the five independent experiments.

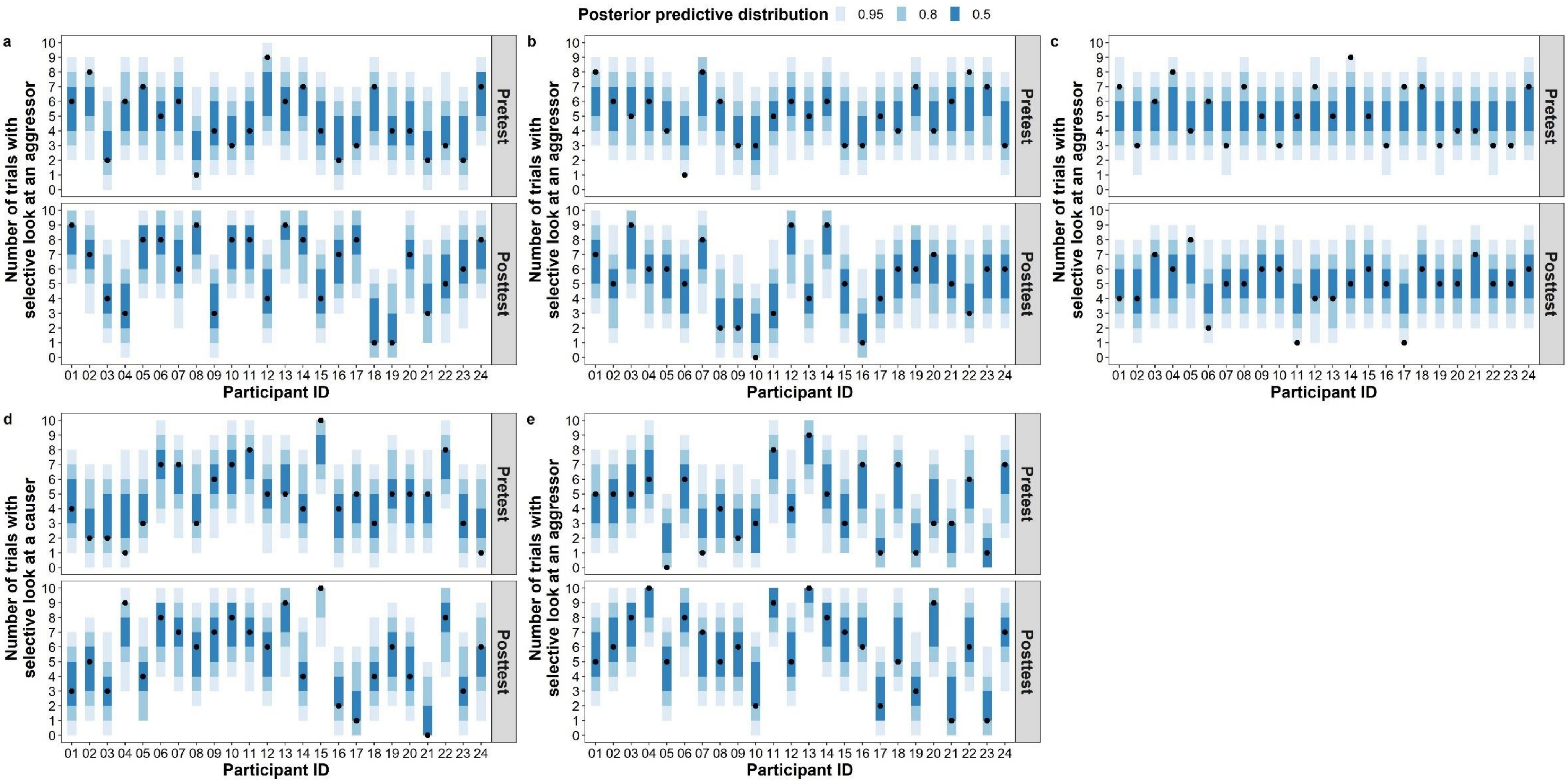

Supplementary Figure 2. Posterior predictive distribution of the number of trials with selective looks towards an aggressor (or a causer) during the pretest or posttest by each participant in Experiments 1 to 5. a-e, we extracted posterior predictive distribution from the best model in model comparison during each experiment (Experiments 1 to 5). The colour of the interval plots corresponds to 95% prediction intervals (light blue), 80% prediction intervals (blue), and 50% prediction intervals (dark blue), respectively. The black dot represents the observed value for each participant.

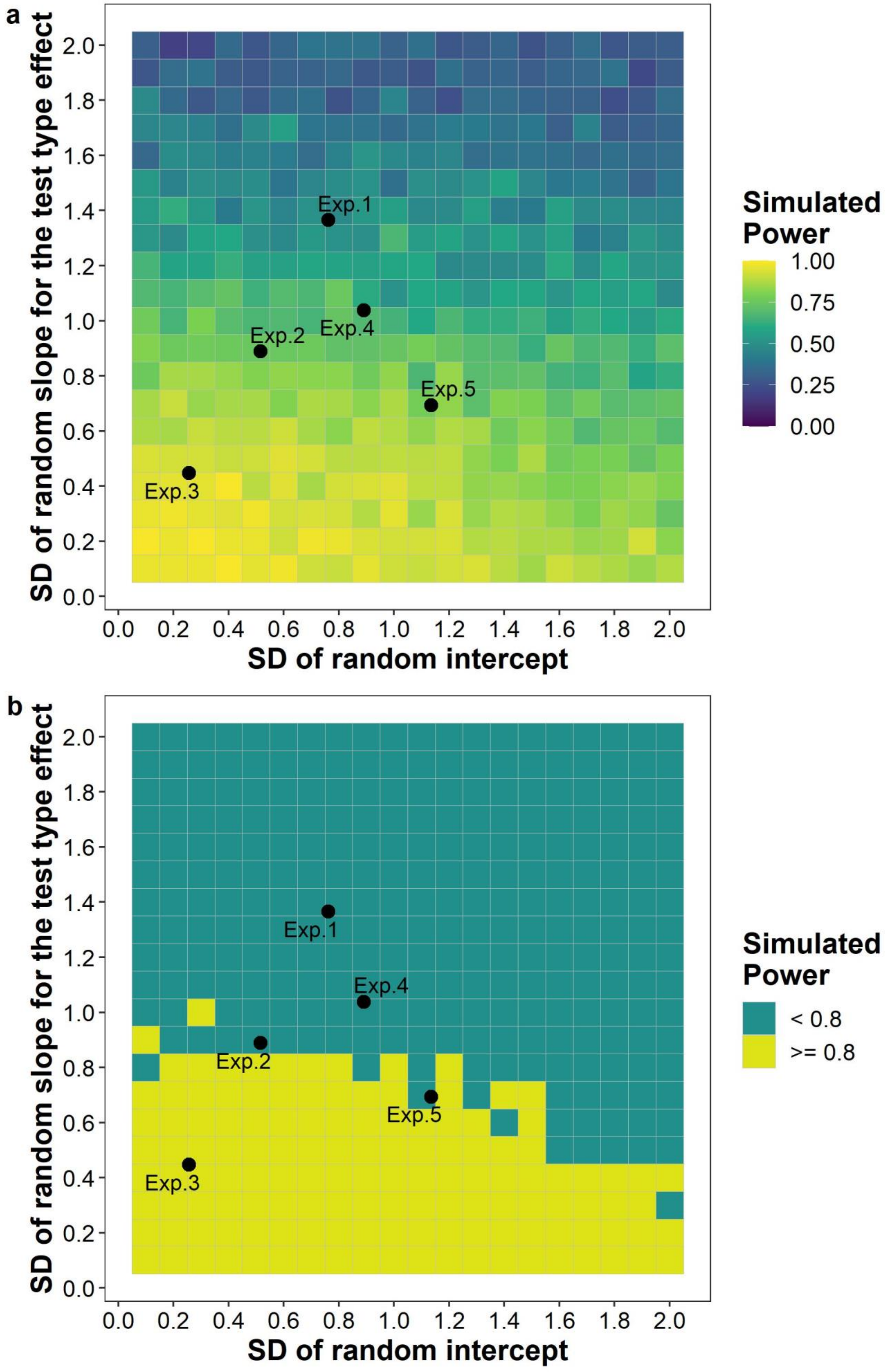

Supplementary Figure 3. Results of simulation-based power analysis based on the actual sample size and

theoretically expected effect size. We randomly generated 100 samples based on a theoretically expected effect size while setting various values for the magnitude of individual difference. Thereafter, we fitted a full model with full random effects structure to the 100 samples to evaluate the extent to which the 95% credible interval of the test type effect did not include zero. The x-axis shows the standard deviation (SD) of random intercept, and the y-axis shows SD of random slope for the test type effect. Each tile represents the combination of SD of random intercept and random slope we set to generate 100 samples. The black dot represents estimated SD of random intercept and SD of random slope for the test type effect in each actual experiment (full model; posterior median). **a**, the colour of the tile represents simulated power in the setting. **b**, the colour of the tile represents whether the simulated power was above 0.8 or not in the setting.

Supplementary Table 1. Parameter estimates, 95% CI, and odds ratios for the best model in the model comparison in Experiment 1.

Effects	Variables	Estimate	MAD	95% CI_{lower}	95% CI_{upper}	Odds Ratio	ESS
Population-Level Effects	(Intercept)	-0.140	0.199	-0.553	0.260	0.869	27971
	**Test type (posttest) [a]**	**0.742**	**0.327**	**0.102**	**1.431**	**2.101**	**21113**
Group-Level Effects	SD of random intercept	0.740	0.244	0.251	1.292	–	11132
	SD of random slope for Test type	1.334	0.334	0.752	2.126	–	11860
	SD of random slope for Trial number	0.561	0.219	0.142	1.061	–	13052
	SD of random slope for Test type : Trial number	0.704	0.333	0.092	1.418	–	13359

Notes: MAD = median absolute deviation of the posterior distribution; CI = credible interval; SD = standard deviation; ESS = effective sample size. Dashes denote values not shown because of their limited interpretation. Posterior medians are shown for odds ratios. The effect of the 95% CI that does not overlap with zero appears in bold.

[a] Test type was dummy coded with the reference category 'pretest'.

Supplementary Table 2. Parameter estimates, 95% CI, and odds ratios for the best model in the model comparison in Experiment 2.

Effects	Variables	Estimate	MAD	95% CI_{lower}	95% CI_{upper}	Odds Ratio	ESS
Population-Level Effects	(Intercept)	0.067	0.152	-0.242	0.381	1.070	42703
Group-Level Effects	SD of random intercept	0.516	0.213	0.090	0.972	–	13869
	SD of random slope for Test type	0.817	0.325	0.160	1.540	–	10922
	SD of random slope for Trial number	0.246	0.179	0.013	0.645	–	20119
	SD of random slope for Test type : Trial number	0.349	0.269	0.019	0.974	–	18492

Notes: MAD = median absolute deviation of the posterior distribution; CI = credible interval; SD = standard deviation; ESS = effective sample size. Dashes denote values not shown because of their limited interpretation. Posterior medians are shown for odds ratios. The effect of the 95% CI that does not overlap with zero appears in bold.

Supplementary Table 3. Parameter estimates, 95% CI, and odds ratios for the best model in the model comparison in Experiment 3.

Effects	Variables	Estimate	MAD	95% CI_{lower}	95% CI_{upper}	Odds Ratio	ESS
Population-Level Effects	(Intercept)	0.020	0.111	-0.201	0.238	1.020	56340
Group-Level Effects	SD of random intercept	0.246	0.192	0.013	0.688	–	14328
	SD of random slope for Test type	0.404	0.287	0.023	1.037	–	11117
	SD of random slope for Trial number	0.271	0.202	0.015	0.736	–	14783
	SD of random slope for Test type : Trial number	0.470	0.307	0.032	1.142	–	11721

Notes: MAD = median absolute deviation of the posterior distribution; CI = credible interval; SD = standard deviation; ESS = effective sample size. Dashes denote values not shown because of their limited interpretation. Posterior medians are shown for odds ratios. The effect of the 95% CI that does not overlap with zero appears in bold.

Supplementary Table 4. Parameter estimates, 95% CI, and odds ratios for the best model in the model comparison in Experiment 4.

Effects	Variables	Estimate	MAD	95% CI_{lower}	95% CI_{upper}	Odds Ratio	ESS
Population-Level Effects	(Intercept)	-0.012	0.211	-0.447	0.427	0.988	25739
Group-Level Effects	SD of random intercept	0.885	0.245	0.465	1.477	–	20909
	SD of random slope for Test type	1.040	0.371	0.316	1.894	–	11964
	SD of random slope for Trial number	0.236	0.197	0.011	0.712	–	17647
	SD of random slope for Test type : Trial number	0.763	0.337	0.109	1.499	–	12201

Notes: MAD = median absolute deviation of the posterior distribution; CI = credible interval; SD = standard deviation; ESS = effective sample size. Dashes denote values not shown because of their limited interpretation. Posterior medians are shown for odds ratios. The effect of the 95% CI that does not overlap with zero appears in bold.

Supplementary Table 5. Parameter estimates, 95% CI, and odds ratios for the best model in the model comparison in Experiment 5.

Effects	Variables	Estimate	MAD	95% CI_{lower}	95% CI_{upper}	Odds Ratio	ESS
Population-Level Effects	(Intercept)	-0.354	0.260	-0.892	0.168	0.702	17341
	**Test type (posttest)** [a]	**0.870**	**0.261**	**0.362**	**1.424**	**2.387**	**41647**
Group-Level Effects	SD of random intercept	1.116	0.255	0.689	1.732	–	18973
	SD of random slope for Test type	0.674	0.397	0.046	1.510	–	8206
	SD of random slope for Trial number	0.395	0.230	0.032	0.901	–	16582
	SD of random slope for Test type : Trial number	0.712	0.380	0.071	1.527	–	9296

Notes: MAD = median absolute deviation of the posterior distribution; CI = credible interval; SD = standard deviation; ESS = effective sample size. Dashes denote values not shown because of their limited interpretation. Posterior medians are shown for odds ratios. The effect of the 95% CI that does not overlap with zero appears in bold.

[a] Test type was dummy coded with the reference category ‘pretest’.

Supplementary Table 6. Comparison of effect size of test type between experiments.

Comparison	Estimate	MAD	95% CI_{lower}	95% CI_{upper}
Experiment 1 − Experiment 2	0.581	0.371	-0.157	1.307
**Experiment 1 − Experiment 3**	**0.756**	**0.367**	**0.030**	**1.498**
Experiment 1 − Experiment 4	0.285	0.370	-0.453	1.023
Experiment 1 − Experiment 5	-0.165	0.370	-0.915	0.568
**Experiment 5 − Experiment 2**	**0.744**	**0.374**	**0.010**	**1.488**
**Experiment 5 − Experiment 3**	**0.920**	**0.371**	**0.196**	**1.656**
Experiment 5 − Experiment 4	0.449	0.374	-0.294	1.198
Experiment 4 − Experiment 2	0.293	0.371	-0.436	1.037
Experiment 4 − Experiment 3	0.468	0.370	-0.253	1.211
Experiment 3 − Experiment 2	-0.175	0.370	-0.904	0.549

Notes: MAD = median absolute deviation of the posterior distribution; CI = credible interval; SD = standard deviation. The effect of the 95% CI that does not overlap with zero appears in bold.

Supplementary Table 7. Mean looking times for each aggressive interaction animation during the movie phase in Experiments 1–5.

	Mean looking time in seconds (standard deviation)			
	**Movie 1**	**Movie 2**	**Movie 3**	**Average**
Exp. 1	16.75 (5.34)	14.81 (6.23)	13.65 (5.93)	15.07 (5.30)
Exp. 2	17.79 (3.20)	17.01 (5.26)	15.01 (6.20)	16.60 (4.49)
Exp. 3	17.78 (3.48)	16.84 (4.01)	16.43 (4.23)	17.02 (3.44)
Exp. 4	16.96 (4.92)	13.79 (4.63)	13.34 (4.65)	14.70 (3.88)
Exp. 5	17.32 (5.03)	16.97 (4.36)	16.17 (4.70)	16.82 (4.35)

Notes: Exp. = experiment.

Supplementary Table 8. Parameter estimates, 95% CI, and odds ratios for the full model assessing test type and trial number effect on selective looks towards an aggressor in Experiment 1.

Effects	Variables	Estimate	MAD	95% CI_{lower}	95% CI_{upper}	Odds Ratio	ESS
Population-Level Effects	(Intercept)	-0.156	0.209	-0.595	0.270	0.855	25432
	Test type (posttest) [a]	0.711	0.353	-0.002	1.452	2.036	18838
	Trial number	0.067	0.190	-0.325	0.469	1.069	27525
	Test type[a] : Trial number	-0.237	0.269	-0.776	0.318	0.789	27330
Group-Level Effects	SD of random intercept	0.762	0.249	0.261	1.315	–	12175
	SD of random slope for Test type	1.365	0.339	0.779	2.148	–	13380
	SD of random slope for Trial number	0.607	0.224	0.185	1.123	–	14768
	SD of random slope for Test type : Trial number	0.708	0.352	0.078	1.465	–	14049

Notes: MAD = median absolute deviation of the posterior distribution; CI = credible interval; SD = standard deviation; ESS = effective sample size. Dashes denote values not shown because of their limited interpretation. Posterior medians are shown for odds ratios.

[a] Test type was dummy coded with the reference category ‘pretest’.

Supplementary Table 9. Parameter estimates, 95% CI, and odds ratios for the full model assessing test type and trial number effect on selective looks towards an aggressor in Experiment 2.

Effects	Variables	Estimate	MAD	95% CI_{lower}	95% CI_{upper}	Odds Ratio	ESS
Population-Level Effects	(Intercept)	0.032	0.172	-0.324	0.383	1.033	43121
	Test type (posttest) [a]	0.026	0.273	-0.537	0.581	1.027	34526
	Trial number	-0.110	0.150	-0.414	0.188	0.896	47737
	Test type[a] : Trial number	0.004	0.223	-0.451	0.447	1.004	44029
Group-Level Effects	SD of random intercept	0.516	0.216	0.088	0.979	–	14540
	SD of random slope for Test type	0.889	0.332	0.219	1.643	–	11303
	SD of random slope for Trial number	0.266	0.190	0.014	0.686	–	17802
	SD of random slope for Test type : Trial number	0.392	0.291	0.020	1.064	–	17191

Notes: MAD = median absolute deviation of the posterior distribution; CI = credible interval; SD = standard deviation; ESS = effective sample size. Dashes denote values not shown because of their limited interpretation. Posterior medians are shown for odds ratios.

[a] Test type was dummy coded with the reference category 'pretest'.

Supplementary Table 10. Parameter estimates, 95% CI, and odds ratios for the full model assessing test type and trial number effect on selective looks towards an aggressor in Experiment 3.

Effects	Variables	Estimate	MAD	95% CI_{lower}	95% CI_{upper}	Odds Ratio	ESS
Population-Level Effects	(Intercept)	0.070	0.148	-0.224	0.368	1.073	44898
	Test type (posttest) [a]	-0.109	0.215	-0.543	0.334	0.897	40658
	Trial number	0.057	0.155	-0.251	0.371	1.059	31277
	Test type[a] : Trial number	-0.036	0.231	-0.504	0.430	0.965	29545
Group-Level Effects	SD of random intercept	0.258	0.199	0.014	0.723	–	12481
	SD of random slope for Test type	0.446	0.302	0.027	1.106	–	9305
	SD of random slope for Trial number	0.309	0.222	0.018	0.813	–	11647
	SD of random slope for Test type : Trial number	0.544	0.328	0.040	1.257	–	10043

Notes: MAD = median absolute deviation of the posterior distribution; CI = credible interval; SD = standard deviation; ESS = effective sample size. Dashes denote values not shown because of their limited interpretation. Posterior medians are shown for odds ratios.

[a] Test type was dummy coded with the reference category 'pretest'.

Supplementary Table 11. Parameter estimates, 95% CI, and odds ratios for the full model assessing test type and trial number effect on selective looks towards a causer in Experiment 4.

Effects	Variables	Estimate	MAD	95% CI_{lower}	95% CI_{upper}	Odds Ratio	ESS
Population-Level Effects	(Intercept)	-0.121	0.230	-0.591	0.362	0.886	27650
	Test type (posttest) [a]	0.385	0.301	-0.242	1.037	1.469	32400
	Trial number	0.042	0.157	-0.274	0.365	1.043	49844
	Test type[a] : Trial number	-0.186	0.275	-0.741	0.393	0.830	38822
Group-Level Effects	SD of random intercept	0.892	0.242	0.476	1.477	—	20816
	SD of random slope for Test type	1.038	0.385	0.290	1.931	—	11340
	SD of random slope for Trial number	0.270	0.218	0.013	0.772	—	17822
	SD of random slope for Test type : Trial number	0.822	0.359	0.120	1.617	—	11323

Notes: MAD = median absolute deviation of the posterior distribution; CI = credible interval; SD = standard deviation; ESS = effective sample size. Dashes denote values not shown because of their limited interpretation. Posterior medians are shown for odds ratios.

[a] Test type was dummy coded with the reference category 'pretest'.

Supplementary Table 12. Parameter estimates, 95% CI, and odds ratios for the full model assessing test type and trial number effect on selective looks towards an aggressor in Experiment 5.

Effects	Variables	Estimate	MAD	95% CI_{lower}	95% CI_{upper}	Odds Ratio	ESS
Population-Level Effects	(Intercept)	-0.386	0.280	-0.969	0.171	0.680	13533
	Test type (posttest) [a]	0.898	0.271	0.362	1.479	2.454	29493
	Trial number	-0.118	0.179	-0.495	0.239	0.888	27406
	Test type[a] : Trial number	0.240	0.275	-0.323	0.821	1.271	29320
Group-Level Effects	SD of random intercept	1.135	0.264	0.702	1.771	–	17173
	SD of random slope for Test type	0.694	0.404	0.050	1.548	–	8722
	SD of random slope for Trial number	0.451	0.238	0.044	0.983	–	15375
	SD of random slope for Test type : Trial number	0.773	0.395	0.073	1.633	–	8362

Notes: MAD = median absolute deviation of the posterior distribution; CI = credible interval; SD = standard deviation; ESS = effective sample size. Dashes denote values not shown because of their limited interpretation. Posterior medians are shown for odds ratios.

[a] Test type was dummy coded with the reference category 'pretest'.

Supplementary Table 13. Parameter estimates, 95% CI, and odds ratios for the model assessing the interaction effect between test type and experiment.

Effects	Variables	Estimate	MAD	95% CI_{lower}	95% CI_{upper}	Odds Ratio	ESS
Population-Level Effects	(Intercept)	-0.147	0.196	-0.541	0.242	0.863	11699
	**Test type (posttest) [a]**	**0.626**	**0.264**	**0.104**	**1.143**	**1.870**	**10899**
	Experiment (Exp.2) [b]	0.203	0.278	-0.343	0.752	1.225	13319
	Experiment (Exp.3) [b]	0.202	0.277	-0.342	0.751	1.224	13874
	Experiment (Exp.4) [b]	0.028	0.276	-0.516	0.581	1.029	13309
	Experiment (Exp.5) [b]	-0.213	0.280	-0.768	0.341	0.808	13744
	Trial number	-0.031	0.052	-0.133	0.072	0.970	32465
	Test type (posttest) [a] : Experiment (Exp.2) [b]	-0.581	0.371	-1.307	0.157	0.559	13395
	**Test type (posttest) [a] : Experiment (Exp.3) [b]**	**-0.756**	**0.367**	**-1.498**	**-0.030**	**0.470**	**12919**
	Test type (posttest) [a] : Experiment (Exp.4) [b]	-0.285	0.370	-1.023	0.453	0.752	13863
	Test type (posttest) [a] : Experiment (Exp.5) [b]	0.165	0.370	-0.568	0.915	1.180	13308
Group-Level Effects	SD of random intercept	0.709	0.100	0.518	0.920	—	13739
	SD of random slope for Test type	0.845	0.145	0.559	1.140	—	7615
	SD of random slope for Trial number	0.283	0.084	0.109	0.444	—	9712

Notes: MAD = median absolute deviation of the posterior distribution; CI = credible interval; Exp. = experiment; SD = standard deviation. Dashes denote values not shown because of their limited interpretation. Posterior median and 95% CI are shown for odds ratios. The effect of the 95% CI that does not overlap with zero appears in bold.

[a] Test type was dummy coded with the reference category ‘pretest’.

[a] Test type was dummy coded with the reference category ‘pretest’.

[b] Experiment was dummy coded with the reference category ‘Experiment 1’.
