# Supplementary material for: Third-party punishment by preverbal infants
Source: Nat Hum Behav. 2022 Jun 9;6(9):1234–42. doi: 10.1038/s41562-022-01354-2 (PMC9489529; doi:10.1038/s41562-022-01354-2)
Supplement: Supplementary file 2 — Reporting Summary [file 41562_2022_1354_MOESM2_ESM.pdf]

nature portfolio

Corresponding author(s): Yasuhiro Kanakogi

Last updated by author(s): March 15, 2022

Reporting Summary

Nature Portfolio wishes to improve the reproducibility of the work that we publish. This form provides structure for consistency and transparency in reporting. For further information on Nature Portfolio policies, see our Editorial Policies and the Editorial Policy Checklist.

Statistics

For all statistical analyses, confirm that the following items are present in the figure legend, table legend, main text, or Methods section.

n/a	Confirmed	
☐	☒	The exact sample size (*n*) for each experimental group/condition, given as a discrete number and unit of measurement
☐	☒	A statement on whether measurements were taken from distinct samples or whether the same sample was measured repeatedly
☐	☒	The statistical test(s) used AND whether they are one- or two-sided<br>*Only common tests should be described solely by name; describe more complex techniques in the Methods section.*
☐	☒	A description of all covariates tested
☐	☒	A description of any assumptions or corrections, such as tests of normality and adjustment for multiple comparisons
☐	☒	A full description of the statistical parameters including central tendency (e.g. means) or other basic estimates (e.g. regression coefficient) AND variation (e.g. standard deviation) or associated estimates of uncertainty (e.g. confidence intervals)
☒	☐	For null hypothesis testing, the test statistic (e.g. *F*, *t*, *r*) with confidence intervals, effect sizes, degrees of freedom and *P* value noted<br>*Give P values as exact values whenever suitable.*
☐	☒	For Bayesian analysis, information on the choice of priors and Markov chain Monte Carlo settings
☐	☒	For hierarchical and complex designs, identification of the appropriate level for tests and full reporting of outcomes
☐	☒	Estimates of effect sizes (e.g. Cohen's *d*, Pearson's *r*), indicating how they were calculated

Our web collection on statistics for biologists contains articles on many of the points above.

Software and code

Policy information about availability of computer code

Data collection	We used customized programming completed in Visual Basic 2015 Express and Tobii SDK (version 2.4.12) to collect data in this study.
Data analysis	Analyses were conducted using the R Statistical language (version 4.0.3; R Core Team, 2020) on Windows 10 x64 (build 19044), using the packages ggpubr (version 0.4.0), effectsize (version 0.5), cowplot (version 1.1.1), cmdstanr (version 0.3.0), tidybayes (version 3.0.0), bayestestR (version 0.11.5), brms (version 2.15.0), BayesFactor (version 0.9.12.4.3), rstan (version 2.26.2) and tidyverse (version 1.3.1). The analysis codes are shared publicly on Github (https://github.com/dororo1225/PunishmentStudy).

For manuscripts utilizing custom algorithms or software that are central to the research but not yet described in published literature, software must be made available to editors and reviewers. We strongly encourage code deposition in a community repository (e.g. GitHub). See the Nature Portfolio guidelines for submitting code & software for further information.

Data

Policy information about availability of data

All manuscripts must include a data availability statement. This statement should provide the following information, where applicable:

- Accession codes, unique identifiers, or web links for publicly available datasets
- A description of any restrictions on data availability
- For clinical datasets or third party data, please ensure that the statement adheres to our policy

The datasets generated and/or analysed during the current study are available on GitHub (https://github.com/dororo1225/PunishmentStudy).

Field-specific reporting

Please select the one below that is the best fit for your research. If you are not sure, read the appropriate sections before making your selection.

☐ Life sciences ☒ Behavioural & social sciences ☐ Ecological, evolutionary & environmental sciences

For a reference copy of the document with all sections, see nature.com/documents/nr-reporting-summary-flat.pdf

Behavioural & social sciences study design

All studies must disclose on these points even when the disclosure is negative.

Study description	The study involved quantitative experimental methodologies.
Research sample	The sample included 24 8-month-olds in each experiment (12 boys and 12 girls in experiment 1, 12 boys and 12 girls in experiment 2, 12 boys and 12 girls in experiment 3, 12 boys and 12 girls in experiment 4, 11 boys and 13 girls in experiment 5). We recruited a developmental sample because our research question pertained to the early emergence of third-party-punishment. The demographics of the sample are included in the methods. The infants in all experiments were from Kanto region (around Tokyo). All infants were of Japanese ethnicity.
Sampling strategy	All samples were convenience samples. Sample size was determined based on prior infant morality studies (Hamlin et al., 2007, 2011; Kanakogi et al., 2013, 2017).
Data collection	The data was all recorded via a Tobii TX300 and regulated by customized programming completed in Visual Basic 2015 Express and Tobii SDK. The participants (infants) were placed on their caregivers' laps during experiments. It was impossible to fully blind the experimenters considering they had to execute the customized programming for different experiments.
Timing	We tested infants from the summer of 2016 to November 2020.
Data exclusions	In experiment 1, 11 infants were tested but excluded owing to distress or fussiness (n = 4) or side-looking bias (n = 7). The criterion of side-looking baias in our study is based on binominal test (see the data analysis). In experiment 2, 18 infants were tested but excluded owing to distress or fussiness (n = 4), experimental error (n = 2), or side-looking bias (n = 12). In experiment 3, 7 infants were tested but excluded owing to distress or fussiness (n = 2), machine trouble (n = 3), or side-looking bias (n = 2). In experiment 4, 17 infants were tested but excluded owing to distress or fussiness (n = 7), machine trouble (n = 1), parental intervention (n = 1) or side-looking bias (n = 8). In experiment 5, 11 infants were tested but excluded owing to distress or fussiness (n = 5), machine trouble (n = 2), or side-looking bias (n = 4).
Non-participation	No participants dropped out/declined participation.
Randomization	The design of each experiment was a one-factor within-participant design, and participants participated in both pretest and posttest. We recruited so that there were 24 participants in each experiment, except for the excluded data. Allocation to each experimental group was random. The measured values were mutually independent across participants and experiments.

Reporting for specific materials, systems and methods

We require information from authors about some types of materials, experimental systems and methods used in many studies. Here, indicate whether each material, system or method listed is relevant to your study. If you are not sure if a list item applies to your research, read the appropriate section before selecting a response.

Materials & experimental systems

n/a	Involved in the study	
☒	☐	Antibodies
☒	☐	Eukaryotic cell lines
☒	☐	Palaeontology and archaeology
☒	☐	Animals and other organisms
☐	☒	Human research participants
☒	☐	Clinical data
☒	☐	Dual use research of concern

Methods

n/a	Involved in the study	
☒	☐	ChIP-seq
☒	☐	Flow cytometry
☒	☐	MRI-based neuroimaging

Human research participants

Policy information about studies involving human research participants

Population characteristics	See above.

Recruitment	Participants were recruited by distributing recruitment flyers at health centers or by registering on the lab website. We contacted all applicants for recruitment. In general, we have very high rates of consent to participate in research and do not suspect any evidence of a problematic self-selection bias in recruitment.
Ethics oversight	Otsuma Women's University's Life Sciences Research Ethics Committee (no. 28-015) and the Behavioral Research Ethics Committee of the Osaka University School of Human Sciences (no. HB020-032)

Note that full information on the approval of the study protocol must also be provided in the manuscript.
